# Confocal LiDAR for remote high-resolution imaging of auto-fluorescence in aquatic media

**DOI:** 10.1038/s41598-023-32036-2

**Published:** 2023-03-23

**Authors:** Joaquim Santos, Peter John Rodrigo, Paul Michael Petersen, Christian Pedersen

**Affiliations:** DTU Electro, Department of Electrical and Photonics Engineering, Technical University of Denmark, Frederiksborgvej 399, 4000 Roskilde, Denmark

**Keywords:** Optical sensors, Applied optics, Imaging and sensing

## Abstract

Spatially resolved in situ monitoring of plankton can provide insights on the impacts of climate change on aquatic ecosystems due to their vital role in the biological carbon pump. However, high-resolution underwater imaging is technically complex and restricted to small close-range volumes with current techniques. Here, we report a novel inelastic scanning confocal light detection and ranging (LiDAR) system for remote underwater volumetric imaging of fluorescent objects. A continuous wave excitation beam is combined with a pinhole in a conjugated detection plane to reject out-of-focus scattering and accomplish near-diffraction limited probe volumes. The combination of bi-directional scanning with remote focusing enables the acquisition of three-dimensional data. We experimentally determine the point spread and axial weighting functions, and demonstrate selective volumetric imaging of obstructed layers through spatial filtering. Finally, we spatially resolve in vivo autofluorescence from sub-millimeter *Acocyclops royi* copepods to demonstrate the applicability of our novel instrument in non-intrusive morphological and spectroscopic studies of aquatic fauna. The proposed system constitutes a unique tool e.g. for profiling chlorophyll distributions and for quantitative studies of zooplankton with reduced interference from intervening scatterers in the water column that degrade the the performance of conventional imaging systems currently in place.

## Introduction

The oceans cover a dominant portion of the Earth’s surface and play a vital role in regulating its climatic systems, thus serving as a key attenuator of climate changes induced by massive discharges of greenhouse gases into the atmosphere. Nonetheless, this regulation comes at the cost of alterations in the oceans’ physical and chemical properties that can threaten local ecosystems and consequently the food security of hundreds of millions of people^1^. In this context, small organisms like zooplankton and phytoplankton are particularly important, since they stand at the base of the prolific marine food chain and as the latter constitutes the largest carbon sink on the planet^2^. Monitoring the biodiversity and biomass of these organisms provides a good indication of the anthropogenic impact on the quality of ecosystems^3^, as their distribution and abundance vary in response to climate change^4^, yet our observational capabilities are still scarce. Over the past years, progress has been made towards the use of photonic and optical technologies to monitor aquatic fauna and flora, in an effort to replace intrusive and laborious manual sampling methods^5^ followed by microscope inspection^6^, and to bridge the coarse resolution and lack of specificity of sonar systems^7^. Optical underwater detection can fundamentally reach the sub-millimeter resolutions required to detect individual organisms, but is challenged by extinction in the propagation medium^8^, which reduces contrast and ultimately imposes the detection limit. In situ imaging techniques are prevailing within the marine ecology and biology community, with several instruments for automated detection of plankton in place^3,9^, such as dark field illumination^10^, imaging flow cytometry^11^, laser-line occlusion^12^, and digital holography^13^. Despite being well-established high-resolution techniques, these are generally restricted to small probe volumes and require the marine particles to either pass through a flow chamber (intrusive) or to be placed at a fixed close-range illuminated region. As a consequence, these devices are usually towed from a vessel and require a dive to produce spatial distributions. Recently, a diver-deployed underwater microscope with an unprecedented resolution of 2.2 μm allowed the observation of the dynamics of coral polyps^14^, but also suffered from narrow short-range inspection volumes.

Because plankton is an extremely diverse group of organisms, spanning from sub-microns up to a meter in size, surveying over various spatiotemporal scales is required to obtain statistically representative descriptions of their distributions^3^. In this sequence, non-intrusive observation methodologies are auspicious as they can unlock remote detection over longer distances and larger volumes^15^, therefore reducing interference with the natural dynamics and behaviors of the organisms in the medium, and having the potential for profiling without the need for a sensor dive, thus saving time and resources. Over the past decades, Light Detection and Ranging (LiDAR) systems have gained interest among the ocean science and engineering communities, having been successfully deployed for local and global remote sensing owing to their deeper penetration into the water column comparing to passive observation platforms^16^, their high accuracy, and the retrieval of vertically-resolved oceanic features with high-spatial density^17^. More concretely, airborne^18,19^ and spaceborne^20,21^ time-of-flight (ToF) LiDAR systems have been typically deployed to investigate, map, and estimate oceanic distributions of plankton. However, due to the long detection ranges, these instruments can only provide crude features and do not resolve single sub-millimeter organisms^15^. Hence, to achieve the desired resolution an in situ platform, deployed either directly in-water or just above the sea surface (e.g. shipborne^22^), is virtually required^17^.

Underwater remote sensing of biota with LiDAR is a relatively new and under-explored field, with the Scheimpflug LiDAR being the most prevalent technology. A non-imaging aquatic Scheimpflug LiDAR combines a powerful collimated blue laser with a tilted 2D detector and dispersive optics to measure elastic and inelastic scattering simultaneously along a line and along a plurality of wavelength channels, with geometrically-encoded range computed using triangulation. Detection of the sub-millimeter copepod species *Daphnia magna* was demonstrated in laboratory using dye-tagged specimens, at distances of more than 4 m^15^. Moreover, fast refresh rates enabled the detection of characteristic oscillatory movements of the pleopods in shrimps^23^. Labelling with fluorescent tags produces much stronger signals comparing to autofluorescence, which is one of the weakest interaction mechanisms that is further accentuated by the high transparency of zooplankton^6^. In a real case scenario, tagging the organisms is not feasible. Phytoplankton exhibits characteristic autofluorescence spectra peaking at around 685 nm, primarily from chlorophyll-*a* molecules^24^. Some zooplankton species also exhibit autofluorescence^25^. Spectroscopic information is an important molecular signature to support taxonomic classification of plankton^26,27^, but is not a sufficient standalone classifier. Morphological information (shape and size) is an essential complementary cue lacking in hyperspectral Scheimpflug LiDARs. A three-dimensional Scheimpflug LiDAR is possible using light-sheet illumination if spectral resolution is sacrificed^28^. Other downside of this LiDAR technology is that a static beam might alter the behavior of photoresponsive organisms^29^.

In the present work, we propose a novel LiDAR platform, which we name inelastic confocal imaging LiDAR, for remote sensing in aquatic media. The optical sensor draws inspiration from the measurement principle of confocal microscopy^30^ to reduce the contributions from scattering elements in the water column that degrade the performance of conventional imaging systems. Our system combines a focused continuous wave (CW) blue excitation Gaussian beam with a matching spatial filtering aperture in a conjugate plane in the detection space. By synchronizing a highly sensitive detector with dual-axis scanning and remote focusing, we obtain images of axially sectioned slices with high sensitivity. By stacking these, we construct volumetric data structures with a high-resolution nearing the diffraction limit. A confocal geometry has been applied in coherent Doppler wind LiDAR^31^ to sense the speed of aerosols in a confined and fixed probe volume. Synthetic aperture confocal imaging allowed to image partially occluded environments, to selectively image different planes, and to further see through water^32^. More recently, confocal diffuse tomography was developed to capture 3D shape through a scattering medium in the elastic channel^33^.

Here we demonstrate for the first time to our knowledge the implementation of remote confocal imaging in an inelastic channel. The system resolution is quantified from empirical axial weighting functions and lateral point spread functions (PSFs). We afterwards acquire an underwater volumetric point cloud with virtual rejection of contributions from intermediary inelastic layers that would otherwise occlude objects of interest in wide field systems, due to the spatial filtering of contributions from out-of-focus planes and scatter. We ultimately show the detection and imaging of laser-induced (i.e. label-free) autofluorescence from a sub-millimeter sized, free-swimming zooplankton species. Our approach is compatible with a variety of high-throughput imaging modalities employed in fluorescence microscopy^34^ and with hyperspectral imaging^35^, making it not only valuable for underwater monitoring applications, but also also attractive to other use cases outside the aquatic medium. A comparison between the proposed method and the current state-of-the-art is summarized in Table 1 for several metrics and aspects of importance in the context of in situ plankton detection.

**Table 1:** Comparison of photonic and optical technologies for in situ plankton detection in several key performance aspects.

Methodology	Non-intrusive?/$z_0$ range	$\textrm{Diving}?^{\text{a}}$	Acquired data	Resolution	Measurement speed	Backscattering suppression?
Laser-line occlusion^12^	$\checkmark$/NA (flow channel + static probe)	$\checkmark$	Binary images (2D morphology)	$\delta x$: 1 mm (particles > 100 μm) 70 mm axial integration	$70 \times 70 \times 1$ mm$^3\,(xyz$) @ 1 MHz	$\times$
Flow cytometry^3,36^	$\checkmark$/NA (flow channel + static probe)	$\checkmark$	2-angle scattering (size) Dual-band fluorescence	$\delta x$: 0.2 μm–100 μm particles	0.2–0.8 mm$^3$/s (12.5–50 mm$^3$/min)	$\times$
Imaging flow cytometry^11^	$\checkmark$/NA (flow channel + static probe)	$\checkmark$	Grayscale images (2D morphology) Side-scattering + chl-a fluorescence	$\delta x$ : 1 μm (5–150 μm particle size) $\delta z$ : 150 μm	4.2 mm$^3$/s (1.5$\cdot$10$^4$ mm$^3$/h)	$\times$
Digital holography^13^	$\times$/450 mm (in water, laser to camera)	$\checkmark$	Holograms (2D morphology)	$\delta x$ : 3.5 μm 450 mm axial recording length	$10.5 \times 7.7 \times 450$ mm$^3$ (*xyz*) @ 25 Hz (max) ($\sim$9.1$\cdot$10$^5$ mm$^3$/s)	$\times$
Dark-field illumination^10,37^	$$\times $$/400 mm (height camera to light sheet)	$$\checkmark $$	Grayscale images (2D morphology)	$$\delta x$$: 73 μm (plankton > 500 μm) 23 mm-thick light sheet	$$\sim 5.4 $$$$\cdot $$10$$^5$$ mm$$^3$$/s (0.7 L/image @ 1.3 Hz)	$$\times $$ (reduction with 90$$\circ $$ illumination)
Underwater microscopy^14^	$\times$/ $\sim$65 mm	$\checkmark$	RGB images (2D morphology & color)	$\delta x$: 2.2 μm (5$\times$)/3.1 μm (3$\times$) $\delta z$ (DoF): 16 μm (5$\times$)/34 μm (3$\times$)	$\sim 0.6$ mm$^3$/s (5$\times$)/$\sim 3.4$ mm$^3$/s (3$\times$) ($2448 \times 2050$ , 3.45 μm pixels @ 15 Hz)	$\times$
Shipborne HSRL^22^	$\times$/Set by extinction ($\sim\,$ 20–30 m typ.)	$\times^{\text{c}}$	Attenuation and backscattering (1D depth profiles)	$\delta z$ : 280 mm	200 mrad (FOV) @ 10 Hz pulse rate (full-profile)	$\times$ (axially-resolved but no filter)
Scheimpflug LiDAR^15,23^	$\times$/Set by geom. or extinction (e.g. 3.5 m–5 m^15^)	$\times^{\text{b}}$	Spectral profiles ($\lambda$) along z-axis (no morphology)	$\delta x$: 12 mm $\delta \lambda$: 5 nm (fast acquisition)	$\sim$8.5$\cdot$ 10$^5$ mm$^3$/s ($\varnothing$12 mm beam, 150 mm axial gate, 50 Hz)	$\times$ (axially-resolved but no filter)
Inelastic confocal LiDAR	$\times$/From $\sim 550$ mm (max set by extinction)	$\times$ (3D probe steer)	Stacked, axially-resolved images (3D morphology, single-channel) 4D acquisition possible ($xyz\lambda$)	$\delta x$: 32 μm @ $z_0$ = 1000 mm $\delta z$: 13 mm @ $z_0$ = 1000 mm $^{\mathrm{c}}$	For reference^d^: $\sim$ 2.3$\cdot$10$^3$ mm$^3$/s (1$^\circ \times 1 ^\circ$ scan @ $z_0$ = 1000 mm) FOV adjustable up to 45$^\circ \times$ 45$^\circ$	$\checkmark$ (confocal imaging)

^a^The instrument needs a vertical dive (e.g. towed from a ship using a rosette sampler) to obtain depth profiles of plankton distributions.

^b^Can only measure profile along a fixed axis (optical axis, *z*). Needs lateral scanning to provide profiles in different transverse coordinates (e.g. relative movement of the ship).

^c^For an 1” objective lens presented herein. Can be enhanced by increasing the exit pupil aperture and decreasing the circular pinhole diameter accordingly.

^d^For a $400 \times 400$ image resolution acquired at 1 Hz. Speed currently limited by the mechanical scanning unit.

The methodologies are considered intrusive if the organisms need to flow through an enclosed probing area inside the instrument in order to be detected. $z_0$ Detection range; $\delta x$ Llateral resolution; $\delta z$ Axial/depth resolution; HSRL High-spectral resolution LiDAR.

## Methods

### Confocal LiDAR system

**Figure 1:**
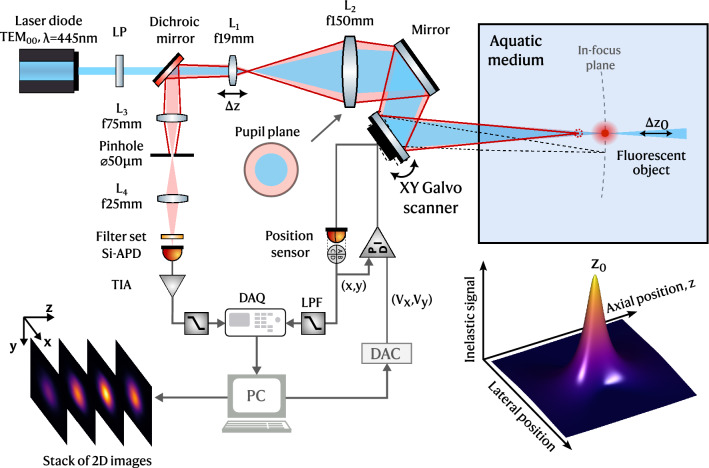
Schematic diagram of the implemented inelastic confocal LiDAR focused at an underwater probing distance $z_0$. A tridimensional representation of the detected signal as a function of the axial and radial (lateral) position relative to the focal plane is depicted.

The schematic diagram of the developed inelastic confocal LiDAR system is shown in Fig. 1. The implemented confocal measurement principle is monostatic by nature, therefore the excitation and emission paths are coaxially aligned, ensuring a consistent field-of-view (FOV) overlap at all distances. This contrasts with underwater bistatic LiDAR architectures^15^ that need to be aligned at a distance, thus with limited overlap between source and receiver that restricts the interaction length (superior and inferior blind range).

We use a compact 445 nm CW Laser Diode (LD, Toptica iBeam-smart 445s) as illumination source to balance the low bulk absorption in water^38^ with an efficient autofluorescence excitation of biological compounds in aquatic biota, as chlorophyll-*a* molecules in algae and phytoplankton^15^. The excitation power through the system is finely adjusted by configuring the laser via software to the maximum setting of 100 mW and using a linear polarizer (LP, Thorlabs WP25M-VIS) as variable attenuator. Since the LD output is a Gaussian beam (single transverse mode TEM$_{00}$) with a quality factor $M^2\sim$ 1.1, we opted not to use an excitation pinhole to reduce the system’s complexity (see Supplement 1 Fig. S1a–c). The collimated excitation plane wave is focused by a pair of achromatic doublet lenses (chromatic and spherical correction) $\text{ L}_1$ (Thorlabs AC127-019-A) and $\text{ L}_2$ (Thorlabs AC254-150-A), with respective focal lengths 19 mm and 150 mm, to a near-diffraction limited single spot at a remote underwater probing distance (or focal plane), $z_0$, measured from lens $\text{ L}_2$. The telescope configuration expands the incoming beam radius to achieve a tighter focus, as the waist radius of the focused Gaussian beam is inversely proportional to the beam radius in the plane of objective lens $\text{ L}_2$ (exit aperture). These pair of lenses was chosen to underfill the back aperture of $\text{ L}_2$ and prevent truncation and diffraction of the beam by any aperture along the optical path. Therefore, untruncated Gaussian beam propagation determines the beam waist and the excitation volume in the probing space. $\text{ L}_1$ is mounted on a resonant piezoelectric linear z-stage (Thorlabs ELL20/M) for automatic mechanical scanning of the probe along the optical axis (*z* axis), i.e. for remote focusing.

The laser-induced fluorescence signal is epi-collected by the full numerical aperture (NA) of $\text{ L}_2$ and collimated by $\text{ L}_1$ upon back-propagation. The inelastic beam is then reflected by a dichroic beamsplitter with 505 nm cut-off wavelength (Thorlabs DMSP505L) to the detection arm of the LiDAR system at 90$^\circ$. Since illumination with a converging spherical wavefront does not selectively illuminate the plane of interest or prevent scattering, a pinhole is needed to spatially filter out-of-focus and scatter light (including background solar light) before the detector and thus accomplish confocal imaging within narrow depth-of-focus (DoF)^30^. With effect, a f75 mm achromatic doublet lens $\text{ L}_3$ (Thorlabs AC127-075-B) focuses the collimated inelastic signal through a 50 μm diameter circular pinhole aperture placed at a plane conjugate to the laser probe, thus ensuring that both the excitation and emission paths are focused on the same spot. The pinhole size was selected to match the near-diffraction limited exp(− 2) waist diameter of the excitation beam, $2\omega _{0b}$, after backpropagation to the pinhole space (i.e. multiplication of $2\omega _{0b}$ by the optical magnification from $\text{ L}_2$ to $\text{ L}_3$, M), to accomplish confocal detection^30^. Because the excitation and emission paths have a common magnification, this condition is consistently met for any probing distance $z_0$. Further opening the aperture leads to a degradation of the axial and lateral resolving power^39^, moving the LiDAR away from a confocal imaging system and toward a wide-field system. The out-of-focus suppression is portrayed in Fig. 1 (solid red line) by ray tracing the inelastic emission from a point source located before the in-focus plane that is focused after the pinhole plane. In this case, only the small amount of photons that do not hit the opaque portion of the pinhole are transmitted, thus having a relatively small contribution to the total integrated signal.

The light transmitted through the pinhole is at last re-imaged by a f25 mm lens $\text{ L}_4$ (Thorlabs AC127-025-AB) onto a silicon avalanche photodiode (Si-APD, Laser Components A-CUBE-S500-01, 1 MHz bandwidth) with integrated transimpedance amplifier (TIA). An emission filter set (FS) matching the fluorophores in the measured specimens selectively transmits the desired wavelength band to the detector and rejects residual elastic scattering reflected by the beamsplitter. A 20 mm diameter dual-axis galvanometer scanner (GS, Thorlabs QS20XY-AG) is included to simultaneously scan the converging excitation beam and de-scan the inelastically backscattered spherical waves emitted from a specimen within the near diffraction-limited probe volume. A 2D fluorescence image over a transverse *xy* plane is constructed pixel-by-pixel through spatial convolution over an axially-selected narrow slice centered at a probing distance $z_0$ by laterally scanning over an underwater FOV. Volumetric imaging (*x*, *y*, *z*) is achieved by stacking the images at different probing distances $z_0$. Since the photons travel time is much smaller than the scanning period, the pinhole is consistently conjugated with the laser probe. Each axis of the GS is controlled by a Proportional-integral-derivative (PID) servo driver. The voltage driving waves ($V_x,V_y$) are generated by a Digital-to-analog Converter (DAC) board (National Instruments USB-6341). An optical position sensor generates a real-time feedback signal proportional to the angular position of its respective mirror, which is used for closed loop operation and to index the detected signal to the respective pixel position within the 3D image matrix. Both the position and APD signals are electronically filtered by first-order low-pass filters (LPFs) and sampled by a Data Acquisition (DAQ) device. A systematic correction factor is introduced computationally to account for relative line delays.

### Measurement setup

For the measurements presented herein we used a $400 \times 400$ mm$^2$ cross-section, 1000 mm-long, 6 mm-thick glass aquarium filled with fresh tap water. The aquarium was placed at a position *z* = $d_{air}$ = 451 mm, with the flat wall orthogonal to the optical axis. The probe was scanned from a distance of 600 mm to about 1200 mm in order to keep the focus underwater. An additional optical magnification factor of $n_w\sim$ 1.33 was introduced upon refraction on the flat air-glass-water interface due to refractive index mismatch, and was accounted in all computations. All distances were measured relative to $\text{ L}_2$ using a rangefinder (Bosch GLM 250VF) for calibration.

In a first stage, the confocal LiDAR resolution was evaluated at seven representative probing distances. The lateral resolution was estimated by experimentally measuring the PSF using 22–27 μm fluorescent microspheres as probes (Cospheric UVPMS-BR-0.995 22–27 μm). The axial resolution was determined by measuring the axial weighting functions of the system with a 1.7 mm-thick fluorescent microscope slide (Thorlabs FSK6). For both directions, the full width at half maximum (FWHM) was considered as the system’s resolution according to the Rayleigh criterion. A total of *N* = 5 measurements were taken along each direction for statistics^40^ and a Richardson-Lucy^41^ deconvolution algorithm with 10 iterations was applied to deduct the effect of the finite probing samples on the raw resolution estimates. A stack of two long-pass filters with 500 nm (Thorlabs FELH0500) and 600 nm (Edmund Optics 62985) cut-on wavelengths were used as emission FS. Subsequently, the system capabilities for volumetric imaging were demonstrated by measuring an artificially-assembled 3D scene with fluorescent objects placed at different z-planes along the optical axis, and processing the data to generate a volumetric render. Finally, the potential application of our underwater confocal LiDAR in remote detection of sub-millimeter aquatic biota in situ and in vivo was evaluated by imaging autofluorescence from samples of the herbivorous zooplankton *Apocyclops royi*. The specimens were kept in brackish water and fed with microalgae prior to the experiment to ensure their guts were filled. A more in-depth description of the experimental procedures is provided in Supplement 1. All data processing and analysis workflow was implemented with custom scripts in Matlab.

### LiDAR system resolution

**Figure 2:**
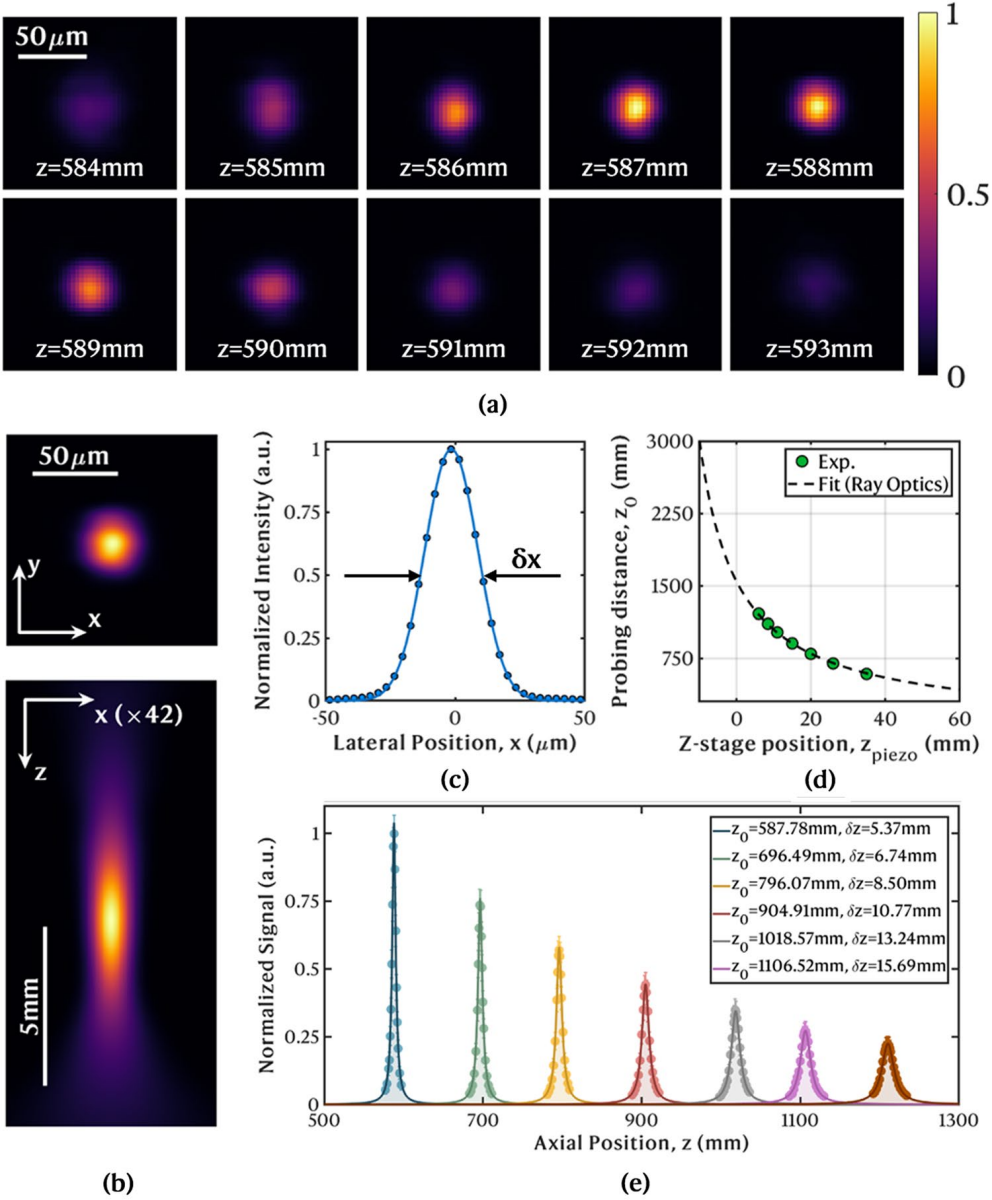
Experimental resolution measurements. (**a**) Montage of the obtained underwater images of an individual 22–27 μm fluorescent microsphere at several z-positions along the excitation probe focused at $z_0 \sim$ 587 mm. Each image has $80 \times 80$ 16-bit pixels acquired with dwell time of 4.44 μs. (**b**) Transverse (*xy*) and axial (*xz*) normalized PSF. For the sake of representation alone, a gridded interpolation was applied (cubic spline in *xy*; Lorentzian interpolant in *z*). (**c**) Associated integrated intensity profile along *x* and respective Gaussian fit with FWHM $\delta x$ $\sim$ 23.93 μm. (**d**) Probing distance, $z_0$, as a function of the $\text{ L}_1$ z-stage position $z_{piezo}$, and imaging described by Eq. (1) using ray optics. (**e**) Deconvolved axial weighting functions at different $z_0$ with Lorentzian fitting curves normalized to the closest range.

Figure 2a shows one raw PSF measurement with 22–27 μm fluorescent microspheres as probes. A montage with multiple frames is displayed for different positions of the bead as it was translated along the optical axis, *z*, for a fixed probing distance $z_0\sim$ 587 mm (of which 136 mm were underwater). The images were acquired with a 4.44 μs dwell time and 1 mW excitation power, which was sufficient to achieve a good Signal-to-noise Ratio (SNR) without photobleaching. Due to the confocal imaging scheme, when the inelastic emitter was located outside the focal plane ($z\ne z_0$), the collected signal was a strongly attenuated version of its peak due to the compound effect of spatial filtering by the pinhole and comparatively lower local power density of the excitation beam outside the waist. The result was a weak and blurred image of the microsphere. As the relative distance to the focal plane decreased, the detected signal emerging from the bead increased, until it peaked when the bead position coincided with the focal plane position ($z=z_0$). At this plane, the diameter of the Gaussian beam was minimum, the transmission of collected fluorescence emission through the pinhole is maximum, and the bead image appeared at its sharpest. The *xy* and *xz* normalized cross-sections of the PSF are depicted in Fig. 2b. From the latter one can visualize the inherent confinement to a small volumetric region around the focal plane in our system. The integrated x-profile from the *xy* PSF is plotted in Fig. 2c. A lateral resolution, $\delta x$, of 23.93 μm was determined from the FWHM of the respective Gaussian fit.

The axial weighting functions measured with the fluorescent slide are traced in Fig. 2e, after deconvolution to correct the effect of the 1.7 mm thickness and normalization to the maximum peak signal. Lorentzian fitting curves are plotted alongside the experimental points. The weighting functions describe, for each probing distance $z_0$, the relative total contribution from inelastic scatterers to the detected signal, according to their relative axial distance to the focal plane, $|z-z_0|$. The axial confinement (optical sectioning) inherent to the confocal architecture is visually perceived through the narrow Lorentzian curves peaking at $z_0$ and quickly decaying as $|z-z_0|$ increases.

Each position of the z-stage holding $\text{ L}_1$ was mapped into a probing distance using ray optics. A plot of measured probing distances, $z_0$, determined from the location of the weighting function peaks, as a function of the z-stage position, $z_{piezo}$, is shown in Fig. 2d. The total travel range of the stage is 60 mm, but only the range from 6 mm (furthest focusing distance) to 35 mm (closest focusing distance) was utilized, restrained by the aquarium dimensions. The data points are plotted alongside an image formation model in the paraxial limit:

1 $$\begin{aligned} z_0 = d_{air} \bigl (1-n_w \bigl ) + n_w \Biggl (\frac{1}{f_2}-\frac{1}{a_o} \Biggl )^{-1} + t_g \Biggl (1-\frac{n_w}{n_g} \Biggl ) \end{aligned}$$

where $f_2$ is the focal length of $\text{ L}_2$, $t_g$ the thickness of the aquarium glass with refractive index $n_g$ $\sim$ 1.52, and $a_o$ is the absolute position of $\text{ L}_2$ relative to the focal plane of $\text{ L}_1$ (point to be imaged). The latter can be decomposed as $a_o = z_{piezo} + \Delta _{ref}$, where $\Delta _{ref}$ is an offset distance set by assembly and determined using the data point for $z_{piezo}$ = 15 mm as reference. This curve describes the LiDAR ranging equation that allows to determine the probing distance for an arbitrary software-adjustable $z_{piezo}$.

The numerical values computed from the resolution measurements at all distances are summarized in Table 2. The disclosed results are the average for the *N* = 5 measurements at each distance. The computed raw $\delta x$ increased from around 24.29 μm at $z_0\sim$ 587 mm to around 40.78 μm at $z_0\sim$ 1210 mm. After correction of the raw estimates with Richardson-Lucy deconvolution, to remove the over-estimation effect due to the 22–27 μm bead sizes, the mean FWHM decreased to about 20.58 μm and 39.05 μm, respectively. The broadening effect became less meaningful as the distance increased and as the bead size became minor relative to the system resolution. For all distances, the ratio $\delta x$ to pixel size was above 6, to ensure an adequate spatial sampling frequency congruent with the Nyquist limit^40^. The axial resolution estimates, $\delta z$, after deconvolution increased from around 5.37 mm to around 18.49 mm with distance. The weighting functions were similarly measured in air for the same $\text{ L}_1$ z-stage positions. From the average ratio between $\delta z$ in water and in air at all distances, a refractive index of $n_w \sim 1.34$ was estimated for the aquatic medium, being close to the reported value at room-temperature and atmospheric pressure^42^.

**Table 2:** Spatial resolution measurements and excitation beam caustics.

${\text{z}_0}$ (mm)	$\delta$x (μm)		$\frac{\delta x}{\mathrm {Pixel \, size}}$	445 nm beam caustics (x)		${\frac{\varnothing \hbox {Pinhole}}{2\omega _{0b}\cdot M}}$	$\delta$z (mm) deconvolved	
	Raw	Deconvolved		Waist FWHM (μm)	$M^2$		Water	Air
587.67 ± 0.12	24.29 ± 1.23	20.58 ± 1.57	6.67	20.85	1.54	0.96	5.37 ± 0.02	3.68 ± 0.09
696.72 ± 0.25	26.30 ± 0.46	23.17 ± 0.54	6.33	24.21	1.47	1.00	6.74 ± 0.03	4.91 ± 0.19
796.13 ± 0.09	29.28 ± 0.98	26.63 ± 1.17	6.37	27.14	1.51	1.03	8.50 ± 0.06	6.23 ± 0.17
904.98 ± 0.20	31.07 ± 0.52	28.47 ± 0.53	7.19	30.89	1.37	1.03	10.77 ± 0.06	7.96 ± 0.06
1018.52 ±0.15	34.87 ± 1.13	32.64 ± 0.53	7.33	33.61	1.34	1.08	13.24 ± 0.10	10.38 ± 0.10
1106.09 ± 0.55	36.84 ± 1.31	34.86 ± 1.20	7.21	36.85	1.41	1.07	15.69 ± 0.09	11.99 ± 0.25
1209.88 ± 0.63	40.78 ± 1.62	39.05 ± 1.57	7.38	39.45	1.46	1.10	18.49 ± 0.06	14.90 ± 0.09

The mean values for *N* = 5 measurements are given with respective deviations. For $\delta z$, only the deconvolved results are included. $\varnothing$Pinhole = 50 μm is the diameter of the circular pinhole aperture and $2\omega _{0b} \cdot M$ is the exp(− 2) excitation beam waist backpropagated to the probe plane.

The excitation beam caustics were experimentally determined at the same z-stage positions, and the obtained FWHM at the waist and $M^2$ are included in Table 2. We observed a degradation of the quality factor from $M^2$ $\sim$ 1.1 at the laser output, to $M^2$ > 1.3, as a consequence of wavefront aberrations introduced as the light propagated through imperfect optical elements (therefore the nomenclature “near-diffraction limited imaging” is used in the manuscript). The empirically-determined ratio between the 50 μm pinhole diameter and the backpropagated exp(− 2) diameter of the excitation beam at the waist was around 1 for all distances. As a consequence of this pinhole aperture choice, the confocal LiDAR resolution, $\delta x$, closely matched the excitation beam FWHM waist diameter. See Supplement 1 for additional calibration details, including the FWHM measurements along the y-axis.

**Figure 3:**
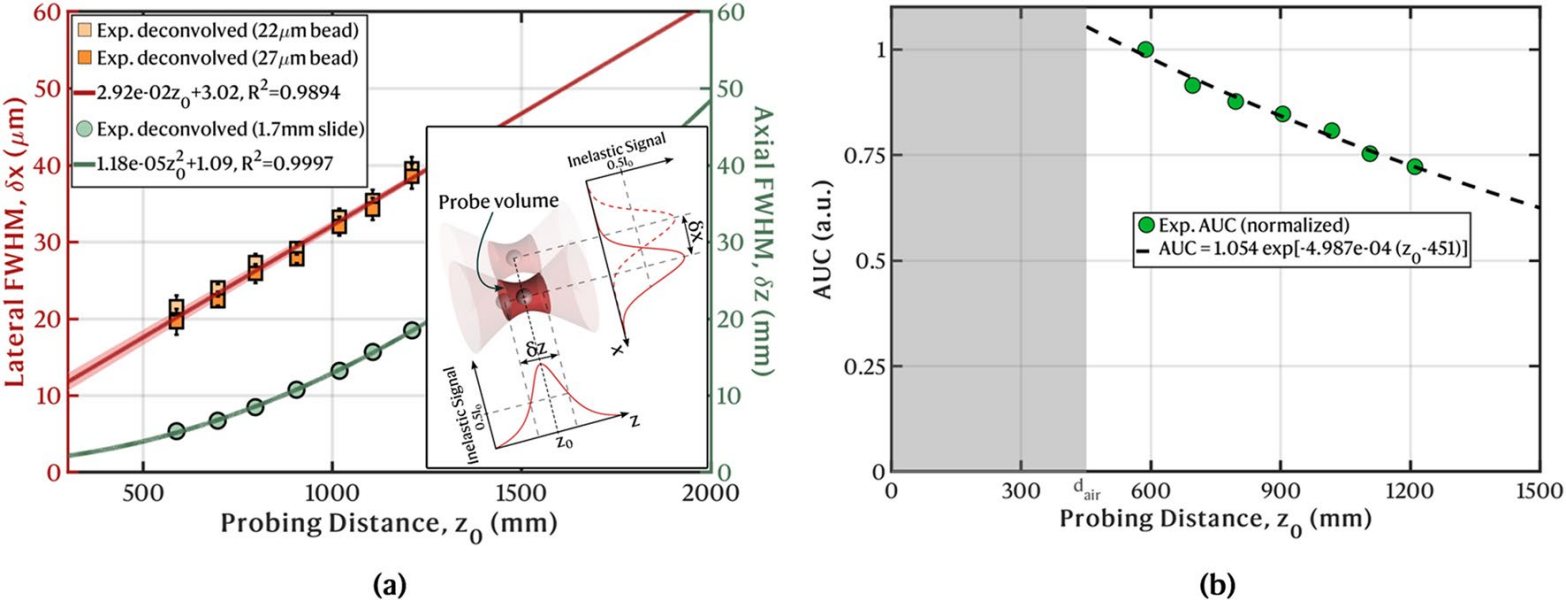
(**a**) Underwater axial ($\delta z$) and lateral ($\delta x$) Richardson-Lucy deconvolved resolutions as a function of the probing distance, $z_0$, with quadratic and linear regressions, respectively. For $\delta x$, the data points from the deconvolution with 22 μm and 27 μm microspheres are plotted to portray the limit cases; the mean is used to determine the fit. Inset: illustration of the probe volume integrated to obtain the intensity of a single pixel. (**b**) Integral of the deconvolved weighting functions (AUC) as a function of the probing distance $z_0$, normalized by the value measured at the closest distance. The datapoints are fitted with an exponential loss function that models the absorption in the underwater optical path $(z_0 - d_{air})$ according to Beer’s law. The shaded region represents propagation in air.

The deconvolved lateral and axial resolutions are plotted in Fig. 3a as a function of the probing distance $z_0$. For the axial resolution, the data points resulting from the deconvolution with both 27 μm and 22 μm diameter microspheres are shown as limit cases, and the mean is considered. An appropriate regression is applied to each set of data points: the lateral resolution $\delta x$ increases linearly with the probing distance; the axial resolution $\delta z$ increases quadratically. The extrapolated resolutions at a distance of 2 m and 5 m are, respectively, $\delta x$ $\sim$ 61 μm $\delta z$ $\sim$ 48 mm and $\delta x$ $\sim$ 150 μm $\delta z$ $\sim$ 296 mm. For each lateral and axial position of the probe the volumetric extent within each the scene under measurement is excited and from which the inelastic scaterrers are integrated to originate a single-pixel intensity is defined by the lateral and axial FWHMs (inset in Fig. 3a). Due to the evolution of these resolutions with distance, the probe volume increases proportionally to $z_0^4$. For all the considered distances, the lateral resolution is well-below the millimeter scale required to detect and image sub-millimeter organisms in water, as for instance zooplankton.

As the probe is scanned to longer underwater distances, the peak signal decreases, and the axial FWHM, $\delta z$, or DoF, increases, resulting in a flattening of the weighing function (Fig. 2e). In the absence of extinction in the optical path, the height of the weighting functions, $H(z_0$), decreases with an inverse quadratic dependence $H(z_0) \propto 1/z_0^2$ due to a reduction in the solid angle sub-intended by the objective lens $\text{ L}_2$. However, the axial FWHM simultaneously increases in a quadratic proportion ($\delta$z $\propto z_0^2$) and, the net result is an approximately constant area under the curve, AUC (energy conservation). Because water is an absorptive medium, there is an exponential energy loss along a total underwater optical path of $\text{2 }(z_0 - d_{air})$. A least squares fitting $\text {AUC}(z_0) = A \cdot \exp {[-\alpha (z_0-d_{air})]}$ (Beer’s law) was thus applied to the integral of the weighting functions in Fig. 2e, where *A* and $\alpha$ are fitting parameters, with the latter representing an estimate of the absorption coefficient of water. The result is plotted in Fig. 3b. An estimated $\alpha$ = $(4.987 \pm 0.763) \times 10^{-4}$ mm$^{-1}$ was computed, which includes the added contributions of the low absorption at the 445 nm excitation wavelength, in the forward direction, and the stronger absorption in the inelastic channel (peaking at approximately 602 nm for the fluorescence slide and extending up to around 750 nm), in the backward direction. This value is of the same order as the reported for pure water^38^, with the excess being likely due to the use of tap water instead. Since the FWHM of the weighting functions was much smaller than the optical path in water for all distances, $\delta z \ll z_0 - d_{air}$, the effect of absorption in the shape of weighting functions was neglected herein.

Additionally, the detection limit was estimated at a distance $z_0 \sim$ 1200 mm ($z_{piezo}$ = 6 mm) by placing a fluorescence slide in the focal plane and gradually decreasing the excitation power through the system until the fluorescence signal detected at the APD matched the noise level. For an excitation power of 34.8 μW at the exit pupil ($\text{ L}_2$) (i.e. prior to reflection at aquarium interface and absorption in water) and a 225 kHz detection bandwidth, the SNR estimated from 500k samples from the APD acquired over a 1 s period was about 0.06 dB. Considering the APD responsivity and transimpedance gain, this translated into an integrated detected power at the detector plane of under 40 pW.

## Results

### Underwater volumetric imaging

**Figure 4:**
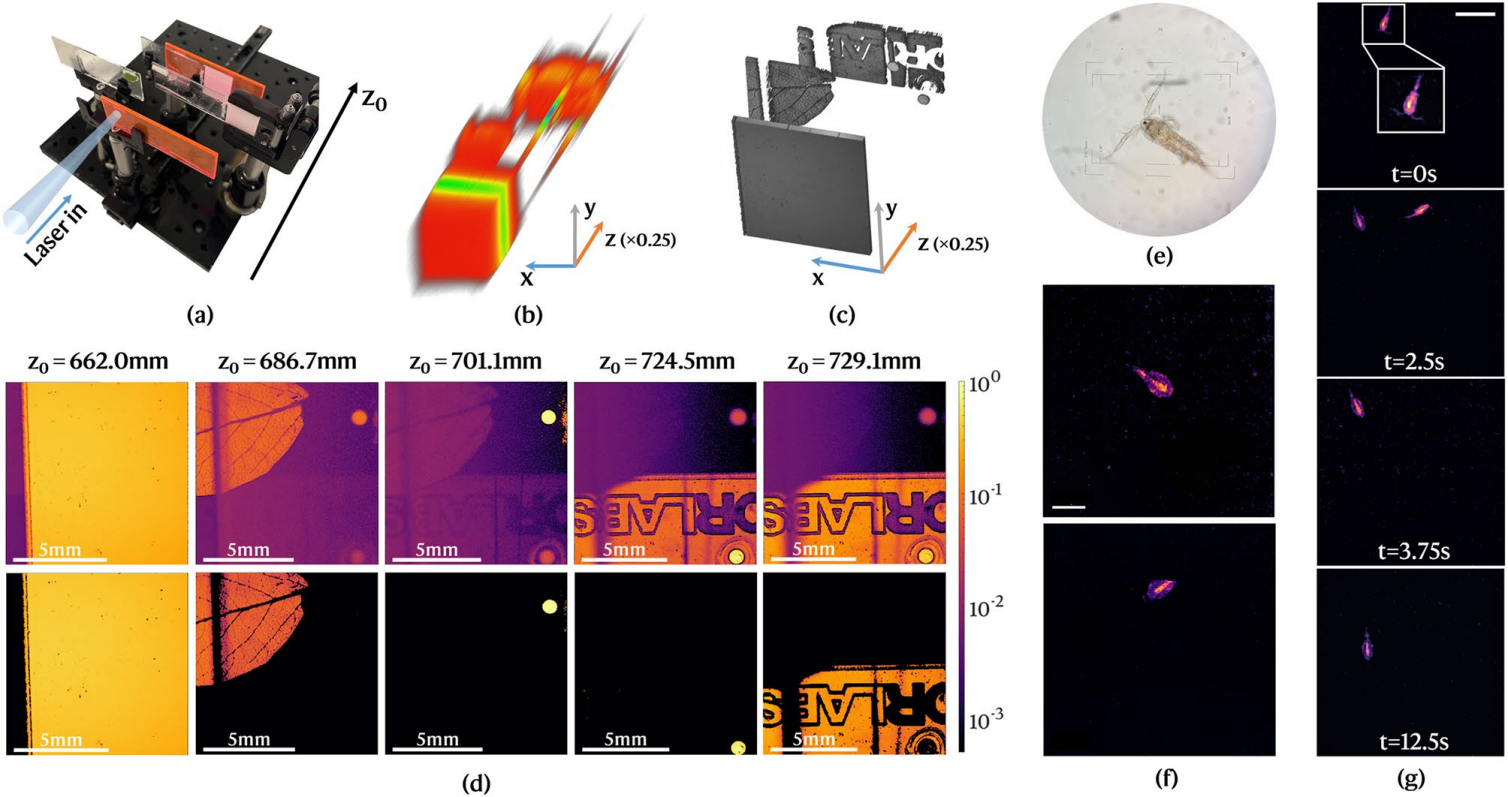
Underwater confocal LiDAR imaging demonstration. (**a**) Photography of the artificial 3D scene assembled. (**b**) 3D point cloud built from the raw stack of confocal images. The z-scale is contracted by a factor of 4. (**c**) Volumetric render of the scene after subtraction of out-of-focus contributions. (**d**) Montage of the best focus images for each layer in a logarithmic fluorescence intensity scale. On the top row, the raw slices; on the bottom, the same slices after correction. Each image has a $600 \times 600$ resolution and 16-bit depth ($\sim$ 7 μs pixel dwell time). (**e**) An Acocyclops royi copepod viewed under a bright-field microscope with 10$\times$ magnification. (**f**) Two examples of underwater autofluorescence images of free-swimming copepods acquired with the LiDAR system at $z_0\sim 587$ mm. $600 \times 600$ image resolution, 5.9 μm pixel size, 5.5 μs dwell time. Scale bar: 500 μm. (**g**) Sequential monitoring of copepods over time. Selection of four $500 \times 500$ illustrative images (9.4 μm pixel size, 5 μs dwell time). Scale bar: 1 mm.

A photo of the scene scanned for the demonstration of volumetric imaging and 3D reconstruction is presented in Fig. 4a. A total of five different layers were assembled with different fluorescent objects along the optical axis: two fluorescent slides, one of which with lettering; a non-fluorescent glass slide with a green leaf; two glass slides with 710–850 μm diameter beads (Cospheric UVPMS-BR-0.995 710–850 μm) and an aggregate of 22–27 μm beads. A total of 183 z-planes were selectively imaged with step-wise z-scanning between around 620 mm and 780 mm, and 1 mW of laser power. The positions of $\text{ L}_1$, $z_{piezo}$, were translated into probing distances $z_0$ using the calibration curve in Fig. 2d. The in-focus slices for each layer, as selected from the 3D image stack, are shown in the top row of Fig. 4d with a logarithmic intensity scale to highlight out-of-focus contributions.

As the probe is z-scanned, the contributions of the unfocused layers can be efficiently suppressed to isolate in-focus fluorescence objects that appear sharp at the intended probing distances. Nevertheless, in the absence of any further processing, out-of-focus objects still contribute to the total signal at each $z_0$ due to the axial roll-off of the Lorentzian weighting functions. This effect can be observed in the raw 3D render in Fig. 4b, in which the objects appear axially extended. The relative contribution is determined by the distance between the z-position of the out-of-focus object and the probing distance, $|z-z_0|$, and $\delta z(z_0)$. This effect can be visualized e.g. in the frame at $z_0$ = 701.1 mm. The large bead in the top right corner. Nevertheless, one can still see the contribution of the autofluorescence from chlorophyll molecules in the leaf, despite more than $10^2$ orders of magnitude below and blurred to such a degree that only the midrib is discernible. If the dynamic range of fluorescence emission among the objects is wide, as is the case for the two previous objects, the absolute contribution of out-of-focus photons might be substantial. This is the case in the images at $z_0$ = 686.7 mm, where the bead located around 14.4 mm away from focus ($|z-z_0|>\delta z$), appears blurred but with a similar intensity level as the in-focus leaf.

In this sequence, the out-of-focus contributions were subtracted from each slice using a pixel-level algorithm described in Supplement 1. The 3D render and the best focus frames after correction are portrayed, respectively, in Fig. 4c-d (bottom row). The confocal detection scheme enables to see through non-opaque fluorescent layers to reveal objects in hidden planes that are apparently obstructed in the inelastic channel, without degradation of the resolution as long as the wavefront is not substantially degraded. In this particular case, the fluorescent slide in the plane closest to the LiDAR system was blocking a major part of the FOV of the sensor, appearing in focus at $z_0$ = 662.0 mm. As the probe was scanned towards longer z-positions it was possible to virtually eliminate its contribution from the other frames shown herein.

### Label-free zooplankton imaging

One possible application of the underwater confocal LiDAR is the remote detection of sub-millimeter aquatic biota in situ and in vivo. We tested the capabilities of our system for remote optical sensing and detection of zooplankton in vivo and in the aquarium. Examples of laser-induced autofluorescence images of the free swimming herbivorous copepod *Apocyclops royi* (shown in Fig. 4e under a bright-field microscope) at a probing distance of $z_0\sim$ 587 mm are shown in Fig. 4f–g. The imaged organisms had a full body length of around 800 μm. A pair of longpass interference filters with cut-on wavelength of 500 nm (Thorlabs FELH0500) was used as emission FS to allow the detection of both the red and cyan autofluorescence bands^25^.

In all the acquired images, a strong fluorescence was detected from the central part of the organisms, likely towards the red and corresponding to undigested chlorophyll content from algae in their guts^25^. Moreover, comparatively weaker bulk fluorescence was detected from the remaining body, with a spatial distribution similar to the cyan autofluorescence detected in Nielsen et al.^25^ for an identical copepod (*Temora longicornis*). This fluorescence might arise from a mixture of flavins, lipopigments, and/or NADH. Since only one inelastic channel was detected, it was not possible to spectrally discriminate the local fluorescence signatures. Nevertheless, the outline of the organisms could be detected and, in some cases, even their antennae (see inset in Fig. 4g). Therefore, the system possesses the resolution required to resolve shape and spatial autofluorescence distributions from individual micrometer-sized specimens, and can provide advantageous morphological information that is not retrieved by e.g. the Scheimpflug LiDAR^15^. Additionally, the system has a high sensitivity and, even though the organisms are highly transparent, a relatively strong signal could be detected at considerably low powers of around 30 mW.

## Discussion and outlook

We have made the first demonstration of an inelastic confocal LiDAR for confined remote sensing in aquatic media. The system can acquire volumetric underwater images with near-diffraction limited resolutions while effectively rejecting out-of-focus light and scatter through spatial filtering. We have shown that the micrometer-order resolution is adequate to detect localized autofluorescence and shape of a free-swimming sub-millimeter sized zooplankton species in vivo, and to determine its positions over time. Both axial and lateral resolutions can be enhanced by increasing the numerical aperture of the excitation beam at the $\varnothing$1” exit pupil. A two-fold increase in the beam diameter in the plane of $\text{ L}_2$ will lead to a decrease in the beam waist diameter at $z_0$ by the same factor, and an axial resolution improvement by a factor of 4. The underwater ranges were limited to a maximum distance $z_0 \sim$1200 mm by the aquarium size but, ultimately, the maximum range will be limited by extinction in water, that depends on its contents^8^. The resolution measurements were performed at low power ($\le$ 5 mW) and a detection limit on the order of tens of pW of detected power was estimated. For the copepods imaging the power was increased to about 30 mW, due to their high transparency. Nevertheless, these relatively low-powers (comparing e.g. with 1 W used in Zhao et al.^15^ that might affect the behaviour and health of microorganisms) reveal the high-sensitivity of the implemented LiDAR system, partially due to the use of a focused probe with higher power densities when compared with collimated illumination systems.

A single inelastic channel was demonstrated in this pilot study, but the LiDAR architecture is compatible with hyper/multispectral imaging. Expanding the number of spectral channels adds a spectroscopic dimension to each pixel, making it possible to acquire 4D point clouds^43^ and acquire unprecedentedly rich morphological and spectroscopic data that potentiates classification tasks. Regarding the temporal resolution, pixel-by-pixel acquisition is an inherently slower process compared with wide-field imaging, and balancing high-speed, high-resolution, high-SNR, and wide FOV/sampling volume is a longstanding challenge^34^. The choice of employing a post-objective scanner to attain wider FOV required the use of a relatively large scanning mirror as a consequence, inevitably leading to lower scanning frequencies. The data throughput in the current system was fundamentally limited by the fast-axis scanning frequency of the GS (and not the electronics) to a maximum of around 200 Hz for small-angles ($\le 1^\circ$). Under these conditions, an 1$^\circ \times$1$^\circ$ FOV at z$_0 \sim$ 1000 mm corresponds to an underwater spatial window of approximately $13 \times 13$ mm$^2$ (considering refraction), acquired with a DoF of $\delta$z $\sim$ 13.2 mm. For reference, if the fast-axis is scanned at a frequency f$_x$ = 200 Hz, then a $400 \times 400$ pixels slice is acquired at an 1 Hz refresh rate (f$_y$ = 0.5 Hz). This translates to a volume sampling speed of around 2.3$\cdot$10$^3$ mm$^3$/s. The inelastic confocal LiDAR can potentially exploit laterally and/or axially parallelized methodologies employed in fluorescence microscopy^34^ (e.g. fast tunable lenses^44^ or line-sheet confocal illumination^45^). Higher laser powers might be used to compensate for smaller pixel dwell times and to achieve the same photon dose delivered to each pixel.

We envision several possible applications for this system in marine or freshwater environments, mainly for short range measurements over distances up to around 5 m. The LiDAR can, for instance, be integrated into a submersible remote operated vehicle (ROV) or deployed in a static platform. One possible application is the profiling of vertical chlorophyll-*a* distributions, e.g. in shallow waters with depths compatible with the previous range. Chlorophyll layers play a central role in marine primary production cycles^46^. For this particular measurement the autofluorescence intensity at around 685 nm^25^ can be used as proxy to indirectly quantify distributions of phytoplankton biomass, and the system might be mounted on a boat pointing downwards into the water column^22^. Owing to the axially-selective imaging, the contribution of intermediate scattering layers can be virtually discarded, but the impact of turbidity on probe volume and penetration depth needs to be studied for different water contents. Furthermore, although the system is incoherent, only background signals from within the DoF will be effectively detected, thus allowing to perform measurements in both shallow and deep waters, both during daytime and night time, provided adequate optical shielding. Alternatively, the laser can be modulated at frequencies up to several tens of MHz and combined with phase-locked detection to further suppress noisy sources. Other possible application is the mapping of interactions, distributions, and movements of zooplankton within the water column^47^. The FOV can be adjusted electronically to zoom-in and out in particular regions of interest to image sub-millimeter organisms or bigger fluorescent specimens. However, the combination of large scanning angles and a flat air-water interface leads to off-axis aberrations that deteriorate the confocal performance, which may be corrected with a glass dome port^48^.

All in all, in the context of underwater remote sensing, there is no one sensor to rule them all, and different surveying methodologies (hyperspectral imaging, camera vision, sonar, and LiDAR) need to be synergistically combined to provide representative sampling of the far-reaching aquatic ecosystems and their dynamics with suitable spatiotemporal resolutions. In this framework, the inelastic confocal LiDAR constitutes a novel imaging and spectroscopic tool for non-intrusive, in situ and in vivo detection of aquatic fauna and flora. As a final remark, the confocal LiDAR imaging system presented here is not restricted to underwater sensing, and it can be also used for terrestrial surveying.

## Supplementary Information

Supplementary Information.

## Data Availability

The datasets generated during and/or analysed during the current study are available from the corresponding author on reasonable request.
