# Supplementary material for: Confocal LiDAR for remote high-resolution imaging of auto-fluorescence in aquatic media
Source: Sci Rep. 2023 Mar 23;13:4807. doi: 10.1038/s41598-023-32036-2 (PMC10036608; doi:10.1038/s41598-023-32036-2)
Supplement: Supplementary file 1 — Supplementary Information. [file 41598_2023_32036_MOESM1_ESM.pdf]

Confocal LiDAR for high-resolution volumetric autofluorescence imaging in aquatic media: supplementary material

JOAQUIM SANTOS[1,*], PETER JOHN RODRIGO[1], PAUL MICHAEL PETERSEN[1], AND CHRISTIAN PEDERSEN[1]

[1] *DTU Electro, Department of Electrical and Photonics Engineering, Technical University of Denmark, Frederiksborgvej 399, 4000 Roskilde, Denmark*
[*] *joasan@dtu.dk*

This document provides information to supplement the manuscript "Confocal LiDAR for high-resolution volumetric autofluorescence imaging in aquatic media".

1. COLLIMATED GAUSSIAN BEAM CHARACTERIZATION

The 445 nm excitation beam outputted by the laser diode (LD) module (Toptica iBeam-smart 445s) was characterized and the results are compiled in Fig. S1a. The beam caustics were measured with a beam profiles (Spiricon M2-200S), and a beam quality factor of $M^2 \leq 1.1$ was obtained from the evolution of the exp(-2) diameter along the beam axis. Due to the high quality of the single-transverse mode beam (TEM_{00}), we opted not to use an excitation pinhole. A cross-section intensity image of the collimated beam is shown in S1b. The beam is slightly asymmetric, which resulted in different M^2 for both directions and a slight astigmatism. The continuous wave (CW) excitation spectrum peaked at $\lambda_0 \approx 449.4$ nm with a full-width at half-maximum (FWHM) $\delta\lambda \approx$ 2.5 nm (measured with an Ocean Optics QEA0262 spectrometer).

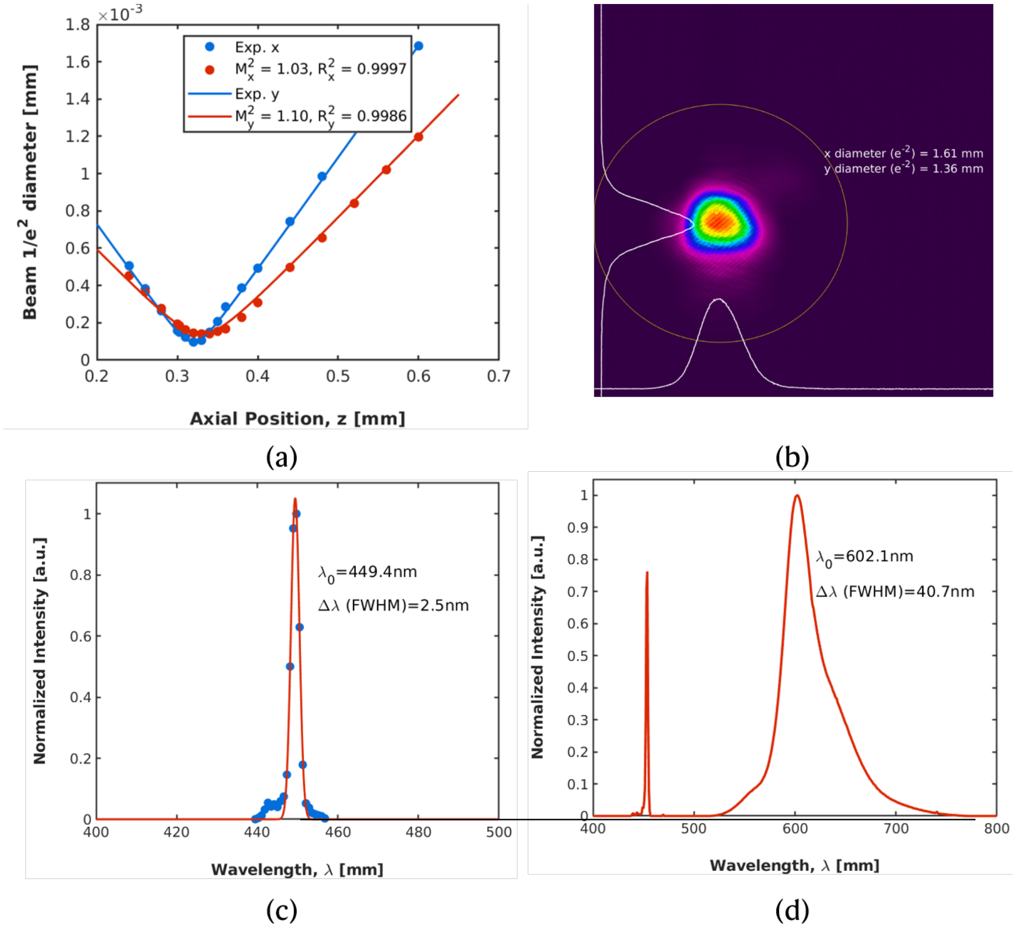

Fig. S1. (a) Excitation Gaussian beam caustics measured from the direct output of the LD module. (b) Cross-section intensity image of the collimated excitation beam, with respective integrated profiles. (c) Spectrum of the excitation light. (d) Autofluorescence emission spectrum of the microscope slide (FSK6) used for the weighting function measurements and 3D reconstruction. The emission is broadband peaking at around 602nm. Note that the narrower and weaker peak at around 450nm corresponded to elastic scattering of the blue excitation beam that is simultaneously detected by the spectrometer. All the laser characterization was performed with the laser in continuous wave (CW) mode, operated at the maximum power of 100mW, to reproduce the operating conditions throughout all the measurements

2. EXPERIMENTAL DETAILS

A. Measurement Setup

The measurements were performed in a 400×400 mm^2 cross-section, 1000 mm-long, 6 mm-thick glass aquarium as a container for the aquatic medium. The one-way air-glass-air transmission (in power) of the flat aquarium wall was around 87% at 445 nm. The z-stage position of L_1 was scanned from z_{piezo} = 35 mm to z_{piezo} = 6 mm, corresponding to probing distances of $z_0 \approx 587$ mm and $z_0 \approx 1210$ mm, respectively (of which, 451 mm were in air).

All 2D images were acquired with bi-directional xy scanning. Both axis of the galvanometer scanner (GS) were driven with triangular waveforms (0.22 V per optical degree). The vertical axis (y) and horizontal axis (x) were set, respectively, as slow and fast axis, and scanned at frequencies f_x and f_y. The frame rate was given by $2f_y$; the frame resolution in pixels by f_x/f_y; the pixel dwell time by $f_y/(2f_x^2)$. The voltage driving waves for the two axis were independently generated by a Digital-to-Analog Converter (DAC) board (National Instruments USB-6341, 840 kHz per channel) with controllable parameters via a custom LabView user interface: scanning waveform, amplitude, phase, and offset.

An electronic zoom could be flexibly adjusted via scanning angle to increase the total measurement volume up to a maximum of $\pm 22.5^o$, however at a cost of speed and resolution. We focused on small angle scanning $\lesssim 1^o$ to achieve a pixel size below the fundamental resolution limit and thus to exploit and characterize the best resolution capabilities of the imaging LiDAR. For these angles, f_x could go up to around 200 Hz. The later frequency was the leading limiting factor to the overall acquisition speeds, and was imposed by mechanical properties as size and weight of the mirrors.

B. Point Spread Functions (Lateral Resolution)

Fluorescent polyethylene microspheres with 22-27 µm diameter (Cospheric UVMPS-BR-0.9995) were assembled on a non-fluorescent microscope glass slide using double-sided tape to ensure the same mounting and immersion medium (i.e. beads directly facing the water), thus avoiding an additional interface. A tweezer was used to pick and sparsely drop the beads on the surface of the slide. Individual beads were located under a bright-field microscope before immersion in water. An additional 710-850 µm fluorescent bead (Cospheric UVPMS-BR-0.995) was used as reference to localize the smaller beads that were invisible to the naked eye (Fig. S2a). The slide was then mounted vertically underwater and perpendicularly to the optical axis using a clamping system attached to a linear stage (Fig. S2b). Several images were acquired at different positions along the fixed probe.

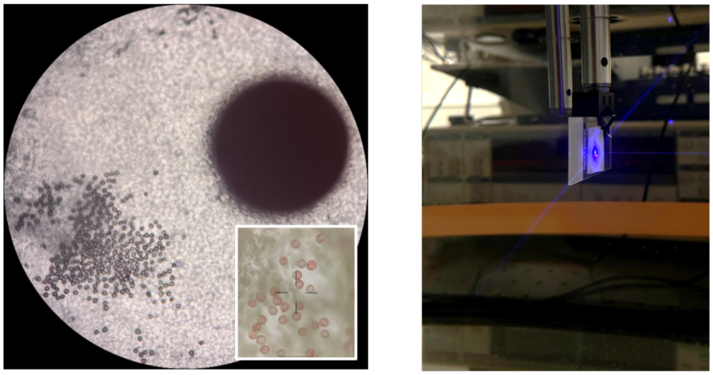

(a) (b)

Fig. S2. Underwater PSF measurements. (a) Image of the 22-27 µm beads under a bright-field microscope with $10\times$ magnification. The large 710-850 µm bead was used for visual localization. The inset shows a zoom-in on the smaller fluorescent beads. (b) Mounting of the samples underwater.

For an accurate estimation of the PSF, the lateral pixel size needs to be at least 2 times smaller than the system resolution in the respective direction, to ensure a sufficient spatial sampling frequency according to the Nyquist theorem [1]. Similarly, detection-wise, and assuming the avalanche photodiode (APD) has sufficient bandwidth, the cut-off frequency of the electronic low-pass filter (LPF) must be equal or greater than the pixel rate (i.e. the inverse of the dwell time), while the Digital Acquisition (DAQ) sampling frequency must be at least twice this value. These considerations ensure that the detection speed can match the scanning speed and the images can be reconstructed properly without blurring induced by under-sampling. The GS was scanned at f_x = 150Hz and f_y = 0.2 Hz, resulting in 750 $\times$ 750 frames with a 4.44 µs pixel dwell time (good signal-to-noise ratio, SNR, and no photobleaching). A 5MHz/channel, 16-bit oscilloscope (Lecroy wavesurfer 104MXs-B) was used as DAQ. The APD signal was filtered by a 225kHz cut-off LPF to minimize the detection bandwidth while respecting the pixel dwell time; the GS position signal was filtered with a 1.5kHz cut-off ($10f_x$). The full-angle Field-of-View (FOV) was adjusted from $0.3^o \times 0.3^o$ to $0.25^o \times 0.25^o$ (in air) as the distance increased. The laser power was similarly increased from 1mW to 5mW with distance, to compensate for the peak intensity changes due to absorption and reduction of the solid angle sub intended by L2, while the APD gain was kept constant. A stack of two long-pass filters with 500 nm (Thorlabs FELH0500) and 600 nm (Edmund Optics 62985) cut-on wavelengths were used as optical filter set (FS).

From the xyz stack of images, individual beads were cropped in 80 $\times$ 80 regions of interest. The

images were filtered with a 5 × 5 Gaussian kernel with σ = 1.25 to reduce scanning artifacts and pixel noise effects. Subsequently, the best focus image was selected and a Gaussian fit applied to the integrated profiles:

$$G(x) = a \exp\left(\frac{-(x-b)^2}{2c^2}\right) \tag{S1}$$

where a, b, and c are fitting parameters. The FWHM of the PSF was used to quantify the system resolution:

$$\delta x = FWHM[G(x)] = 2c\sqrt{2\ln 2} \tag{S2}$$

The effect of the bead size as deducted by deconvolving the raw PSF with a 2D Gaussian kernel with full-width at tenth-maximum of both 22 µm and 27 µm to account for the beads size distributions. A Richardson-Lucy [2] algorithm with 10 iterations was applied and the deconvolved FWHM was determined as previously from the new integrated profiles. For most distances considered herein, the effect of the bead size in the resolution estimates was not negligible since its diameter was not sufficiently small to be approximated by a δ-Dirac. Ideally, the beads diameter should have been sub-resolution to straightforwardly provide an accurate estimation of the system resolution [1], but smaller beads would mean a more intricate manipulation and preparation for the underwater measurements. This procedure was repeated for N=5 microspheres for each probing distance, and descriptive statistics was applied.

C. Weighting Functions (Axial resolution)

A fluorescent microscope slide (Thorlabs FSK6) with broadband autofluorescence (Fig. S1d) was mounted orthogonally to the optical axis, and the axial intensity profiles were measured by translating the slide along a static probe focused at z_0 (i.e. no imaging) in 1mm steps. At each position, a total of 16-bit 5× 100k samples of the APD signal were acquired (National Instruments USB-6341 as DAQ), and averaged to provide each experimental point. For each z_0, the signal was plotted as a function of the slide position along the z-axis. A Lorentzian fit was applied [3]:

$$\phi(z) = \frac{H(z_0)}{1+\left(\frac{z-z_0}{\delta z}\right)^2} \tag{S3}$$

where $H(z_0)$ if the height and δz the FWHM fo the weighting function, and therefore the axial resolution or DoF. The laser power through the system was set to 1mW and the APD gain was adjusted to prevent saturation at the closest distance. The conditions were kept constant for all probing distances to allow direct comparison between the measurements at different distances (universal normalization), and thus to evaluate the effect of absorption in water. Richardson-Lucy deconvolution was applied using a square pulse function with 1.7 mm width.

D. Label-free Zooplankton Imaging

A culture of *Apocyclops royi* was kept in brackish water and fed with microalgae. A few specimens were then transferred to a water-filled glass cuvette using a pipette. The recipient was mounted underwater at the focal plane of the laser at $z_0 \approx 587$ mm (z_{piezo} = 35 mm). Multiple sets of contiguous frames ($\geq$ 10) was then acquired continuously. The filter set was change to a pair of long-pass filters with cut-on wavelength of 500 nm (Thorlabs FELH0500), to allow the simultaneous detection of both cyan and red autofluorescence [4]. An excitation power of around 30 mW was used to obtain a sufficient SNR as the organisms were semi-transparent (small absorption). All images were processed as follows: 1) subtraction of the background offset; 2) filtering with 5×5 median kernel for pixel noise reduction; 3) unsharp masking to compensate smoothing by amplifying high-frequency components; 4) Richardson-Lucy deconvolution with 10 iterations and the experimental PSF; 5) adaptive histogram equalization to further enhance image contrast.

3. ADDITIONAL NUMERICAL RESULTS

Table S1. Numerical results obtained from the PSF measurements. δy represents the lateral FWHM resolution along the vertical axis.

z_{piezo} (mm)	z_0 (mm)	FOV (air) (^{o})	Pixel size (µm)	δx (µm)				δy (µm)				FWHM/Pixel size	
				Raw	Dec. (22µm bead)	Dec. (27µm bead)	Dec. (mean)	Raw	Dec. (22µm bead)	Dec. (27µm bead)	Dec. (mean)	x	y
35	587.67 ± 0.12		3.08	24.29 ± 1.23	21.56 ± 1.47	19.60 ± 1.68	20.58 ± 1.57	24.16 ± 0.58	21.11 ± 0.93	19.01 ± 1.15	20.06 ± 1.04	6.67	6.50
26	696.72 ±0.25	0.3 × 0.3	3.66	26.30 ± 0.46	24.02 ± 0.50	22.32 ± 0.57	23.17 ± 0.54	27.01 ± 1.17	24.89 ± 1.33	23.35 ± 1.54	24.12 ± 1.44	6.33	6.59
20	796.13 ±0.09		4.18	29.28 ± 0.98	27.35 ± 1.11	25.91 ± 1.24	26.63 ± 1.17	29.77 ± 1.01	27.94 ± 1.21	26.66 ± 1.44	27.30 ± 1.33	6.37	6.53
15	904.98 ±0.20		3.96	31.07 ± 0.52	29.14 ± 0.50	27.81 ± 0.56	28.47 ± 0.53	30.80 ± 1.13	28.55 ± 1.53	26.73 ± 2.17	27.64 ± 1.85	7.19	6.98
11	1018.52 ±0.15	0.25 × 0.25	4.46	34.87 ± 1.13	33.22 ± 1.17	32.07 ± 1.23	32.64 ± 0.53	34.35 ± 1.23	32.53 ± 1.19	31.30 ± 1.56	31.92 ± 1.37	7.33	7.16
8.5	1106.09 ± 0.55		4.84	36.84 ± 1.31	35.40 ± 1.38	34.31 ± 1.43	34.86 ± 1.20	36.80 ± 1.21	34.93 ± 1.34	33.49 ± 1.46	34.21 ± 1.40	7.21	7.07
6	1209.88 ± 0.63		5.29	40.78 ± 1.62	39.54 ± 1.55	38.56 ± 1.60	39.05 ± 1.57	41.46 ± 3.04	40.29 ± 3.15	39.31 ± 3.36	39.80 ± 3.25	7.38	7.52

Table S2. Further experimental results obtained from the beam caustics and weighting functions. $2\omega_{0,b}$ is the excitation beam exp(-2) diameter at the waist and $2z_R$ twice its Rayleigh length, which is multiplied by the estimated refractive index of water ($n_w \approx 1.34$) to correct for the change in wavelength with the medium. $M_{probe\rightarrow pinhole}$ is the total optical magnification from the probe plane to the pinhole space.

z_0 (mm)	445nm beam caustics (x)				$M_{probe\rightarrow pinhole}$	$\frac{\varnothing \mathbf{Pinhole}}{2\omega_{0b}\cdot \mathbf{M}}$	δz (mm)			
	FWHM waist (µm)	$2\omega_{0,b}$ (µm)	M^2	$\mathbf{2z_R \cdot n_w}$ (mm)			Raw	Dec. (1.7mm slide)	Air	$\mathbf{n}_w$
587.67 ± 0.12	24.29 ± 1.23	21.56 ± 1.47	1.54	5.89	1.47	0.96	5.65 ± 0.01	5.37 ± 0.02	3.68 ± 0.09	
696.72 ±0.25	26.30 ± 0.46	24.02 ± 0.50	1.47	7.94	1.22	1.00	6.96 ± 0.03	6.74 ± 0.03	4.91 ± 0.19	
796.13 ±0.09	29.28 ± 0.98	27.35 ± 1.11	1.51	9.98	1.06	1.03	8.67 ± 0.06	8.50 ± 0.06	6.23 ± 0.17	
904.98 ±0.20	31.07 ± 0.52	29.14 ± 0.50	1.37	12.93	0.92	1.03	10.90 ± 0.06	10.77 ± 0.06	7.96 ± 0.06	1.34 ± 0.07
1018.52 ±0.15	34.87 ± 1.13	33.22 ± 1.17	1.34	15.30	0.81	1.08	13.35 ± 0.10	13.24 ± 0.10	10.38 ± 0.10	
1106.09 ± 0.55	36.84 ± 1.31	35.40 ± 1.38	1.41	18.39	0.75	1.07	15.78 ± 0.09	15.69 ± 0.09	11.99 ± 0.25	
1209.88 ± 0.63	40.78 ± 1.62	39.54 ± 1.55	1.46	21.08	0.68	1.10	18.57 ± 0.06	18.49 ± 0.06	14.90 ± 0.09	

4. SUBTRACTION OF OUT-OF-FOCUS CONTRIBUTIONS FROM THE 3D IMAGES

This section expands on the procedure employed to subtract out-of-focus contributions from the volumetric image of the scene pictured in Fig. 3a in the manuscript, and that resulted in the 3D render shown in Figure 3c.

From the stack of 183 images at different probing distance z_0, the in-focus image for each layer was selected (shown again in Fig. S3a with a logarithmic intensity scale). Due to the axial extension of the Lorentzian functions at the selected distances (Fig. S3b), out-of-focus objects will contribute to all the frames with different relative weights. For instance, when the focal plane of the LiDAR system coincides with the plane of the leaf (z_0 = 686.7 mm, plotted in orange in Fig. S3b), the relative distance to the first bead (at z = 701.1 mm, plotted in green) dictates that the latter will have a relatively small contribution of around 5% of that of the in-focus leaf. Nevertheless, in absolute terms, this translates into an intensity that is comparable to the fluorescence emission from chlorophyll in the leaf, as the bead is a much stronger inelastic scatterer.

The following procedure was implemented in MATLAB at the pixel level:

1. Application of a Savitzky-Golay smoothing filter [5] with a 3rd order polynomial and frame length of 9 along the z-axis, to correct pixel noise (Fig. S3c for representative pixels).

2. Detection of local maxima along the z-axis, i.e. the planes at which an object is in focus at the considered pixel-level (marked with crosses in Fig. S3c bottom), using a prominence threshold to avoid noisy determinations.

3. Evaluation of contiguous frames to determine if they belong to a thick, as fluorescence can both arise from superficial layers or from the bulk of a thick, semi-transparent object.

4. Dispensation of the non-selected frames/z-planes as they do not contain relevant information or in-focus objects (i.e. all the information arises from out-of-focus contributions).

5. Subtraction of contributions from out-of-focus objects in the selected non-contiguous frames. Since the location of the objects is known, z_{peak}, as well as its peak intensity, $I(x, y, z_{peak})$, the absolute contribution at any slice can be computed and subtracted:

$$\hat{I}(x, y, z_0) = I(x, y, z_0) - I(x, y, z_{peak}) \frac{1}{1 + (\frac{z_0 - z_{peak}}{\delta z})^2} \frac{H(z_{peak})}{H(z_0)} \tag{S4}$$

   The second term on the right-hand side of the equation corresponds to the peak pixel intensity weighted by the relative displacement to the probing distance, z_0, and the relative height of the weighting function (correct water absorption and change in the solid angle).

6. Thresholding to eliminate reminiscent noise

7. Filtering with 3×3 median kernel followed by unsharp mask

8. Rendering of the 3D point cloud with Matlab's Volume Viewer app. A comparison of the 3D rendering of the raw data and the correct data is shown in Fig. S3d.

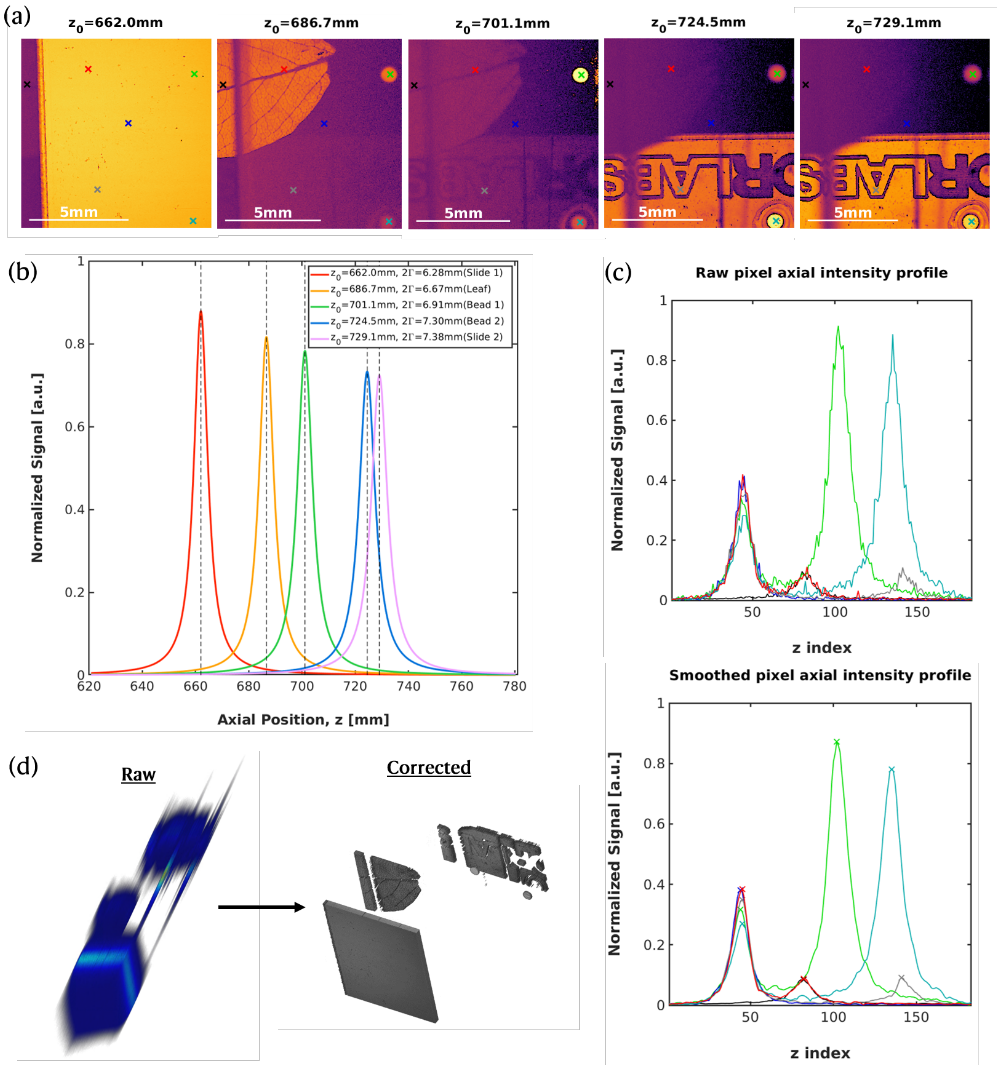

Fig. S3. Subtraction of out-of-focus contributions from the underwater volumetric images. (a) Montage of the best focus images for each layer with a logarithmic intensity scale. (b) Weighting functions at the selected distances. (c) Axial intensity profiles of representative pixels signaled in (a). On top, the raw profiles; on the bottom, the profiles after application of a 3rd oder Savitzky-Golay filter. The change in the captured features is negligible. (d) 3D render of the raw data and respective result after correction.

REFERENCES

1. R. W. Cole, T. Jinadasa, and C. M. Brown, "Measuring and interpreting point spread functions to determine confocal microscope resolution and ensure quality control," Nat. Protoc. **6**, 1929–1941 (2011).
2. D. A. Fish, J. G. Walker, A. M. Brinicombe, and E. R. Pike, "Blind deconvolution by means of the Richardson–Lucy algorithm," J. Opt. Soc. Am. A **12**, 58 (1995).
3. L. Meng, C. Pedersen, and P. J. Rodrigo, "Cw direct detection lidar with a large dynamic range of wind speed sensing in a remote and spatially confined volume," Remote. Sens. **13** (2021).
4. J. H. Nielsen, C. Pedersen, T. Kiørboe, T. Nikolajsen, M. Brydegaard, and P. J. Rodrigo, "Investigation of autofluorescence in zooplankton for use in classification of larval salmon lice," Appl. Opt. **58**, 7022–7027 (2019).
5. W. H. Press and S. A. Teukolsky, "Savitzky-Golay Smoothing Filters," Comput. Phys. **4**, 669 (1990).
